# The “Suicide Guard Rail”: a minimal structural intervention in hospitals reduces suicide jumps

**DOI:** 10.1186/1756-0500-5-408

**Published:** 2012-08-04

**Authors:** Andreas Mohl, Niklaus Stulz, Andrea Martin, Franz Eigenmann, Urs Hepp, Jürg Hüsler, Jürg H Beer

**Affiliations:** 1 Psychiatric Services Aargau AG/Teaching Hospital of the University of Zurich, Haselstrasse 1, P.O. Box 1044, Baden, CH-5401, Switzerland; 2 Department of Medicine, Cantonal Hospital of Baden/Teaching Hospital of the University of Bern and the University of Zurich, Baden, CH-5404, Switzerland; 3 Clinic and Policlinic for Anaesthesiology and Pain Therapy, Bern University Hospital, BHH F-230, Bern, CH-3010, Switzerland; 4 Institute of Mathematical Statistics and Actuarial Science, University of Bern, Sidlerstrasse 5, Bern, CH-3012, Switzerland; 5 Department of Medicine, Bern University Hospital, Bern, CH-3010, Switzerland

## Abstract

**Background:**

Jumping from heights is a readily available and lethal method of suicide. This study examined the effectiveness of a minimal structural intervention in preventing suicide jumps at a Swiss general teaching hospital. Following a series of suicide jumps out of the hospital’s windows, a metal guard rail was installed at each window of the high-rise building.

**Results:**

In the 114 months prior to the installation of the metal guard rail, 10 suicides by jumping out of the hospital’s windows occurred among 119,269 inpatients. This figure was significantly reduced to 2 fatal incidents among 104,435 inpatients treated during the 78 months immediately following the installation of the rails at the hospital’s windows (*χ*^2^ = 4.34, df = 1, p = .037).

**Conclusions:**

Even a minimal structural intervention might prevent suicide jumps in a general hospital. Further work is needed to examine the effectiveness of minimal structural interventions in preventing suicide jumps.

## Background

Jumping from heights is a readily available and lethal method of suicide accounting for about 10% of suicides in Switzerland [1]. The introduction of barriers at specific sites which have acquired publicity for suicide attempts (*hot spots*) has been shown to reduce suicide attempts or deaths by jumping [2,3]. Measures to prevent jumps at notorious suicide sites range from barriers that make jumps impossible (e.g., security nets) [2] to minimal interventions which are more of a psychological barrier (e.g., telephone helplines at the hot spot) [4]. Findings show that the majority of survivors of suicide jumps do not go on to commit suicide [5,6] and that the restriction of access to means of suicide may prevent suicides [7]. This suggests that it is worthwhile making preventive efforts at jumping hot spots in order to reduce overall suicide rates.

**Figure 1:**
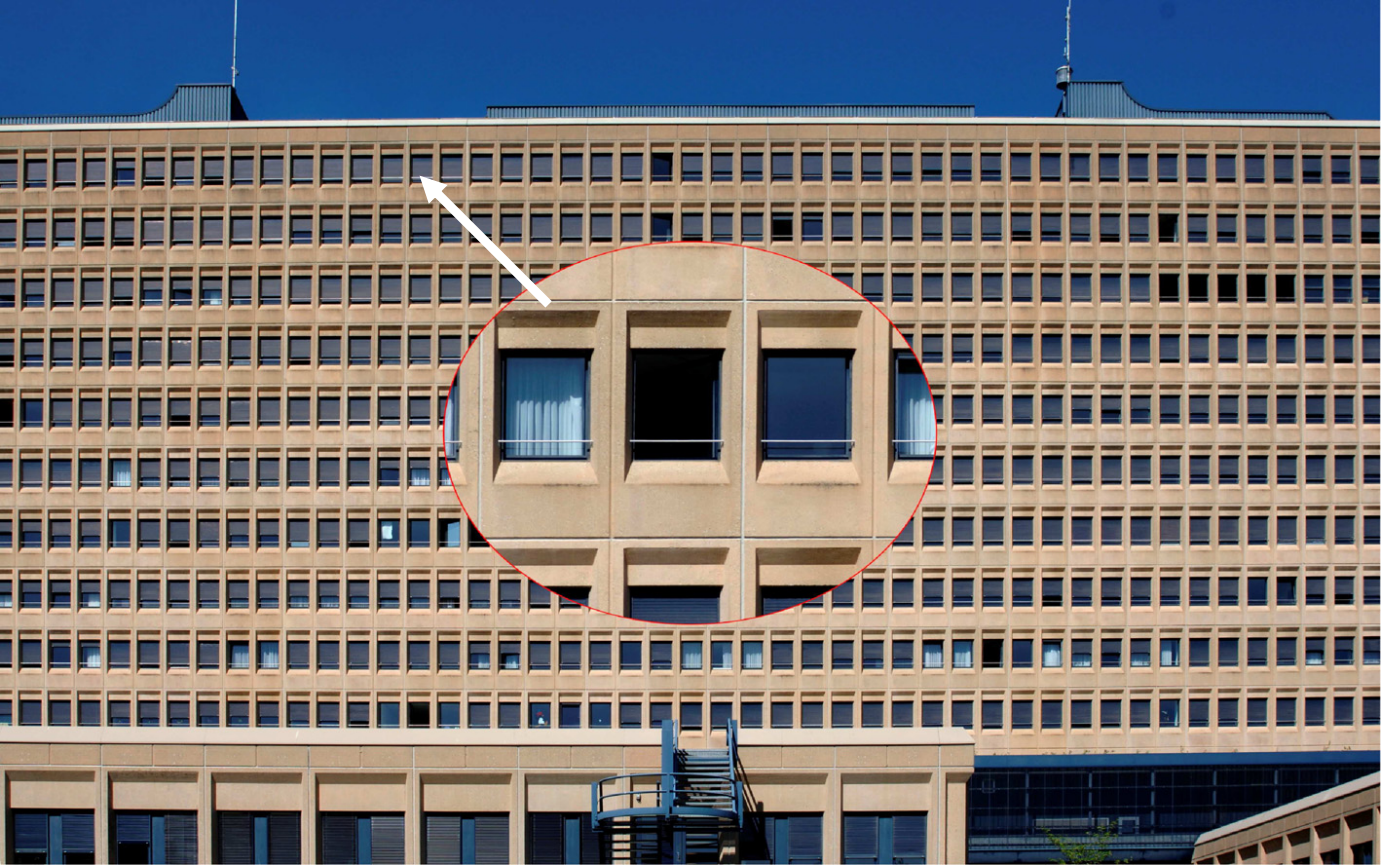
The metal rail installed at each of the 1’240 hospital windows.

The Cantonal Hospital in Baden is a Swiss general teaching hospital located in a high-rise building and has approximately 370 beds. During the past decades the hospital was confronted with a significant number of fatal jumps out of the hospital’s windows. These incidents happened despite an action plan implemented to prevent suicides. This action plan included the continuous training of physicians and nurses in the recognition of the signs and symptoms of suicidal behaviour, a well-equipped consultation and liaison psychiatric service and the temporary seclusion of high-risk patients in secured rooms with locked windows, etc. Due to the limited effects of this action plan [8], it was decided that, commencing June 2004, a 20 mm diameter metal guard rail would be installed at each of the 1,240 hospital windows to discourage suicidal patients from jumping out of the windows (Figure 1). This metal rail was placed at a height of 113 cm, 18 cm above the window sill. Although most people could climb over this rail with relative ease, it was hypothesized that the rail represented at least some sort of psychological barrier which might deter patients from spontaneous suicidal action.

The aim of this study was to examine whether there was a reduction in the number of suicide jumps following the installation of this minimal structural intervention.

## Methods

In order to compare the number of suicide jumps prior to and following the installation of a minimal structural intervention at the windows of the Cantonal Hospital of Baden, police records and patient charts from the hospital for 16 years (January 1995 to December 2010) were reviewed. This study was approved by the hospital’s Internal Review Board.

We used *χ*^*2*^-statistics, controlling for (a) the number of cases treated in the hospital, and (b) the number of inpatient days before and after the intervention in order to evaluate the difference in the number of suicide jumps prior to and following the installation of a metal guard rail at every hospital window.

## Results

Between January 1995 and December 2010, 12 fatal incidents involving falls from windows occurred at the hospital. 10 patients (mean age: 61 years; range: 31–80 years) committed suicide by jumping out of a window before the metal guard rails were installed in June 2004 (114 months or 9.5 years; 119,269 inpatient cases, 1,029,962 inpatient days). Two fatal falls (mean age: 73 years; 70 and 75 years) happened after their installation (78 months or 6.5 years; 104,435 inpatient cases, 796,926 inpatient days). Controlling for the number of inpatient cases, this corresponds to a statistically significant reduction of suicide jumps following the installation of the metal rails (*χ*^*2*^ = 4.34, *df* = 1, *p* = .037). Weighted for the number of inpatient days, the reduction in suicides nearly reaches statistical significance (*χ*^*2*^ = 3.55, *df* = 1, *p* = .06). Note: there were no further non-fatal suicide jumps after the installation of the metal rails.

One of the two fatal post-intervention cases was a disorientated and delirious woman (70 years old) who accidentally fell out of the window at night. The second post-intervention case was a non-patient male visitor (75 years old) who had obviously conceived a plan to commit suicide at this hospital some years earlier when his wife died there of cancer.

## Discussion

This study suggests the effectiveness of a minimal structural intervention in preventing suicide jumps in a general teaching hospital. Controlling for the number of inpatient cases, there was a statistically significant reduction of suicide jumps following the installation of a metal guard rail at each window of the high-rise building. The number of inpatient cases is probably the most valid indicator for estimating the population at risk of committing suicide as the majority of general hospital suicides occurs within the first few days after admission [9]. When weighting for the number of inpatient days, the reduction in suicide jumps reached nearby statistical significance (p = .06). Considering that suicide is a rare event, such marginal cases can also be considered effective if there is a clear-cut reduction in the absolute number of incidents [10]. Our finding is in line with previous research demonstrating reduced suicide falls when safety barriers (e.g., security nets) are installed at known suicide jumping sites [2,3]. Given the impulsive nature of many suicidal acts and the often short-term nature of acute suicidal crises [11,12], such preventive measures might even reduce overall suicide rates, at least to some degree [13]. This is supported by findings from previous research which indicate that there seems to be no immediate and complete shift to other jumping sites [2] or to other methods [7,13]. One important exception to this is the study by Sinyor & Levitt [14]: after securing a bridge in Toronto they actually found there was a shift to other nearby bridges. In our particular case a complete shift to other nearby jumping sites is rather unlikely as there are no other high-rise buildings or other heights in the vicinity of the hospital, and most inpatients at this general hospital are, to some extent, immobile. If one assumes that suicidal crises in general hospitals often follow the disclosure of a severe somatic diagnosis, or are associated with a severe medical condition, and that these crises are generally of a temporary nature, the prevention of short-term suicidal impulses might actually lead to the prevention of the suicides themselves.

Regarding the two fatal falls which occurred during the post-intervention phase of our study, one must take into account that they were not preventable. One was an accident with no suicidal intention; the other resulted from a long-term planned action and lacked any spontaneous component. The specific circumstances of these two fatal falls following the installation of the metal guard rail at the hospital windows provide further support for our hypothesis of a reduction in the number of suicide jumps following the minimal structural intervention.

It is interesting to note that although the metal rails made jumping out of the windows somewhat more difficult, for most patients they probably did not present an insurmountable physical barrier. Rather, the rails seem to represent a visual barrier which inhibits spontaneous suicidal behaviour psychologically. The impact of non-structural interventions (e.g., hotlines at suicide hot spots) in preventing suicides has been demonstrated in earlier studies [4,15]. Such findings merit attention as the full structural protection of many suicide spots is simply not possible, or only at a prohibitive cost.

The metal rail may nevertheless also constitute a real physical barrier for some of the hospital patients who are typically aged and frail. These patients require more time and preparatory work to assume a jumping position and this, in turn, may provide valuable time for reflection and so hinder impulsive behaviour. One could also hypothesize that the additional time gained due to the presence of the metal rail may prove crucial to those attempting to rescue suicidal patients before they jump.

Among the limitations of this study is the lack of a control group which, strictly speaking, precludes a causal interpretation of the findings. For ethical reasons the lack of a control group is inherent in almost all studies evaluating suicide prevention measures at hot spots. Gunnell & Frankel [16] state that in well-conducted randomized controlled trials, no specific intervention has been shown to reduce suicides. Bearing this in mind, we can only try to exclude alternative explanations. In our case the hospital capacity and resources did not change substantially over the study period; in fact, there was a decrease in the mean duration of treatment at the hospital during the past years (1995: M = 9.0 inpatient days vs. 2010: M = 7.0 inpatient days). This is in line with a general trend observed throughout Switzerland and is not necessarily an indication of less severe cases. On the contrary, the hospital is a central hospital for a region with approximately 300,000 inhabitants and in the period observed there was recorded a tendency to more severe cases [17]. Furthermore, despite a declining overall suicide rate in Switzerland during the past decades (after reaching a peak in the late 1970s/early 1980s), suicides by falls have not followed that trend and remained more-or-less constant (at about 2 incidents per 100,000 people) in recent years [1] (unpublished data: Swiss cause-of-death statistics, courtesy of the Federal Statistical Office in Neuchatel).

Another limitation of the study concerns the unknown extent to which this preventive measure led to an overall reduction in suicide rates. We know that there was no suicide by other means at the hospital during the post-intervention period but we do not know whether there were patients who postponed their suicidal intention and subsequently committed suicide after being discharged from the hospital. This data is not available in Switzerland due to data protection laws. Method substitution inevitably occurs in some cases where access to a particular suicide method is restricted, but a complete shift to other methods or jumping sites is unlikely. For example, after securing a well-known jumping site in Bern/Switzerland, suicides ceased completely at this place and there was no shift to other nearby jumping sites [2]. We know that many survivors of suicide attempts do not go on to commit suicide [5,6]. Nevertheless, we have to acknowledge that measures to limit the availability of the means of suicide are aimed mainly at reducing those suicidal acts that are impulsive or which are the result of an acute or temporary crisis [18].

## Conclusions

While needing further replication, the findings of the study at least suggest that even with minimal structural interventions suicide jumps might be prevented. The results also indicate that barriers at jumping sites in hospitals might not only prevent falls among suicide jumpers who typically suffer from psychiatric disorders [5], but also among general hospital patients who are predominately diagnosed with somatic disorders.

## Competing interests

The authors declare that they have no competing interests.

## Authors’ contributions

AM1, AM2, FE, and JHB designed the study. AM1, AM2, FE, and JHB were involved in the data collection. NS and JH performed the statistical analyses. AM1, NS, UH, and JHB were involved in the interpretation of the data. AM1, AM2, FE, and JHB drafted the manuscript. AM1, NS, UH, JHB reviewed the manuscript several times. All authors have read and approved the final version of the manuscript.
